# Transcranial fully endoscopic clipping techniques for ruptured aneurysms: an initial experience from a single center

**DOI:** 10.3389/fneur.2026.1705373

**Published:** 2026-03-05

**Authors:** Zhengxing Xie, Chengyang Xie, Yan Zhuang, Jieping Liu

**Affiliations:** 1 Department of Neurosurgery, The Affiliated Hospital of Jiangsu University, Zhenjiang, China; 2 Neuro-Endoscope and Mini-Invasive Treatment Center, The Affiliated Hospital of Jiangsu University, Zhenjiang, China; 3 Jiamusi University, Jiamusi, China

**Keywords:** aneurysm clipping, endoscopic surgery, fully endoscopic technique, indoor surgery mode, ruptured aneurysm

## Abstract

**Background:**

Ruptured intracranial aneurysms remain a formidable challenge in neurosurgery. This study presents our initial experience with fully endoscopic bimanual clipping for ruptured aneurysms and aims to evaluate its safety and feasibility.

**Methods:**

In this retrospective single-center study, patients with ruptured aneurysms scheduled for clipping underwent surgery exclusively via fully endoscopic bimanual techniques. The data collected included patient records, radiological images, aneurysm characteristics, surgical details, and postoperative outcomes.

**Results:**

From January 2022 to April 2025, eight consecutive patients with ruptured aneurysms (two women and six men, with a mean age of 64.1 ± 10.2 years) underwent fully endoscopic clipping. Aneurysm locations included the middle cerebral artery (*n* = 1), posterior communicating artery (*n* = 1), anterior communicating artery (*n* = 3), anterior cerebral artery (*n* = 2), and ophthalmic artery (*n* = 1). No intraprocedural rupture occurred. Endoscopic inspection and postoperative computed tomography angiography confirmed complete aneurysm occlusion with preservation of all parent and perforating vessels in all cases. No postoperative cerebral infarctions attributable to vessel compromise were observed. No mortality related to the endoscopic procedure was observed. During follow-up ranging from 1 to 28 months, seven patients (87.5%) achieved excellent or good recovery (Karnofsky Performance Status [KPS] ≥ 80), and one patient showed improved KPS.

**Conclusion:**

Fully endoscopic bimanual clipping for ruptured aneurysms is safe and feasible. This technique provides enhanced visual information for intraoperative decision-making while minimizing unnecessary tissue manipulation and retraction. Accumulating experience suggests that this technique holds promise for further improving the quality of surgical care for ruptured aneurysms.

## Introduction

Although significant progress has been made in vascular neurosurgery, ruptured intracranial aneurysms continue to represent a catastrophic emergency, posing substantial challenges owing to brain swelling, obscured subarachnoid spaces, and the risk of re-rupture (1, 2). Although endovascular techniques have advanced, surgical clipping remains a cornerstone of management and cannot be entirely replaced by endovascular methods (3–5). However, persistent concerns regarding residual aneurysm filling and parent vessel compromise underscore the need to refine conventional clipping techniques.

Recently, increasing experiences with the application of endoscopy have documented promising results in trigeminal neuralgia (6), meningioma (7), and pituitary adenoma (8). Growing evidence has documented the advantages of endoscopy, such as brighter illumination, panoramic view, and finer detection of anatomical details, which shed new light on many conventional neurosurgeries and improve the outcome of the patients. Some authors term the fully endoscopic techniques as “indoor surgery mode,” which contrasts with the “outside door mode” used in conventional microscopic view (7). Compared to “outside door mode” neurosurgery using a microscope, the “indoor surgery mode” enables neurosurgeons to look around the corner of the surgical field and perform more precise manipulation. Although neurosurgeons have paid increasing attention to fully endoscopic techniques, little is documented about their application in aneurysm clipping, particularly in the clipping of ruptured aneurysms. Whether a fully endoscopic clipping of a ruptured aneurysm is safe and feasible remains to be determined.

This study aims to bridge the gap in the literature by retrospectively analyzing eight ruptured aneurysms using fully endoscopic clipping techniques. We seek to elucidate the safety and feasibility of fully endoscopic clipping of aneurysms, providing valuable insights to improve surgical techniques and enhance the overall efficacy of endoscopic surgery.

## Methods

This study was approved by the Research Ethics Committee of the Affiliated Hospital of Jiangsu University (Zhenjiang, China). Informed consent was obtained from all enrolled patients. The inclusion criteria were a diagnosis of ruptured aneurysm, no other contraindication for surgery, and an aneurysm combined with an intraparenchymal hematoma. The exclusion criteria were unruptured aneurysm, recurrent aneurysm, and heavy calcification at the aneurysm neck, which cannot be treated by simple clipping surgery.

Eight consecutive patients with ruptured aneurysms underwent fully endoscopic clipping from January 2022 to April 2025 in the Department of Neurosurgery at an affiliated hospital of Jiangsu University. All data, including age, sex, clinical symptoms, imaging data, Hunt-Hess grade, Fisher grade, intraoperative time, Karnofsky performance status (KPS) score before and after surgical treatment, surgical records, and videos, were reviewed. All patients underwent a computed tomography scan, which demonstrated subarachnoid hemorrhage (SAH) or hematoma. Subsequently, computed tomography angiography (CTA) of the brain was performed, and a ruptured aneurysm was diagnosed. Neurological examinations were performed in all patients. Intraprocedural rupture (also referred to as premature rupture in this study), defined as rupture before complete aneurysm dissection and control, was recorded as a hazardous event of aneurysm clipping.

### Surgical procedure

A multidisciplinary team (MDT) discussion, including interventional radiologists of our department, radiologists, anesthetists, and neurosurgeons, was conducted to determine the relative risks and benefits of clipping versus endovascular treatment. We also took the patients’ preferences into consideration before making the final decision for clipping surgery. If applicable, we preferred one of the following approaches: lateral supra-orbit, supraorbital keyhole, pterional, or unilateral interhemispheric. For patients with low H-H grades and Fisher grades, we preferred minimally invasive keyhole craniotomy; for those with high H-H grades and Fisher grades, we preferred conventional craniotomy. Once the craniotomy was completed, a 0-degree endoscope (4 mm diameter, 18 cm length; Karl Storz, Germany) was introduced into the surgical field, and all the surgical procedures were performed under endoscopic view, without any assistance from a microscope. The endoscope was held using a mechanical holder (Karl Storz, Germany) to facilitate bimanual dexterity. The surgical corridor was primarily established through gravity assistance and cerebrospinal fluid drainage by opening the cisterns, typically without the use of fixed retractors. If additional access was needed, a ventricular puncture or opening of the lamina terminalis was performed. For patients with a hematoma, we usually evacuated part of the clots first to reduce intracranial pressure. Unlike conventional microscopic clipping, the retractorless technique was regularly used during our fully endoscopic clipping surgery (also referred to as endoscope control surgery in this study). Bimanual microsurgical techniques were applied to open the arachnoid, dissect and identify the architecture of the aneurysm, and clip the aneurysms exclusively under endoscopic view. The entire procedure can be grouped into three stages: the dissection and identification stage before clipping, the clipping stage, and the evaluation of clip position stage. In the first stage, approaching observation and a panoramic view of the endoscope facilitate neurosurgeons to recognize the relationship between the aneurysm and the parent, branching, and perforating arteries, as well as adjacent structures with less manipulation of the aneurysms and parent artery, which significantly reduces the risk of intra-procedural rupture. If blind spots exist, we use a 30/45 degree endoscope to check for such blind spots. Proximal control was performed on a regular basis to facilitate relaxation and further dissection of the aneurysm. Sometimes, in order to facilitate the dissection and detection of high-risk spots before the final clipping, we also performed temporary partial obliteration of the aneurysm to secure the rupture site. After gaining full recognition of the architecture of the aneurysm, the final clipping was performed. After the placement of the clip, its position was further evaluated via endoscopy to confirm the completeness of aneurysm obliteration while preventing the parent artery, its branches, and perforators from occlusion or constriction. During this stage, we also punctured and coagulated the aneurysm to facilitate the detection of the clip position. If any compromise was indicated, the clip was repositioned under endoscopic view to achieve an optimal placement. All patients underwent CTA before discharge to confirm the complete obliteration of the aneurysm. Additionally, CT/MRI scans were performed to discover any potential infarctions after the procedure. All patients were followed up regularly after discharge at the 1st, 3rd, and 6th months, respectively, and then annually thereafter.

## Results

In our series, eight ruptured aneurysms were successfully clipped via fully endoscopic techniques at our department from January 2022 to April 2025. The clinical features of all patients are described in Table 1. Among these eight patients, there were two women and six men, and their ages ranged from 45 to 75 years (64.1 ± 10.2 years). Preoperative CTA confirmed one middle cerebral artery aneurysm, one posterior communicating artery aneurysm, three anterior cerebral communicating artery aneurysms, two anterior cerebral aneurysms, and one ophthalmic aneurysm. All patients presented with subarachnoid hemorrhage, and three patients harbored intraparenchymal hematomas. Among the eight patients, two were classified as grade II on the Hunt and Hess (H&H) scale, four were classified as grade III, and two were classified as grade IV upon admission. The patient characteristics are listed in Table 1. According to the Fisher scale, one patient was grouped into grade 2, four were grouped into grade 3, and three were grouped into grade 4. The pterional approach was used in two patients, the lateral supraorbital approach was used in three patients, and the supraorbital keyhole approach was used in one patient. The unilateral interhemispheric approach was utilized for two aneurysms. A minimally invasive keyhole craniotomy was performed in three patients. All the aneurysms were dissected and clipped successfully without intraprocedural rupture using fully endoscopic bimanual techniques. The retractorless technique and proximal control were applied for all patients. Temporary partial clipping was performed in one patient with anterior cerebral aneurysm to facilitate the further dissection of the rupture spot from the adherent brain tissue. During the endoscopic inspection after clipping, complete occlusion of the aneurysm was observed along with the preservation of the parent, branching, and perforating vessels, which were also confirmed by postoperative CTA. The mean intraoperative time was 148.3 ± 28.3 min. No procedure-related deaths or complications directly attributable to the endoscopic technique were observed. The follow-up period ranged from 1 to 28 months. Seven patients (87.5%) demonstrated excellent or good recoveries (KPS≧80). The remaining patient showed improved KPS. Hydrocephalus occurred in one patient 1 month after clipping and they underwent a VP shunt. Our experience indicated that the fully endoscopic bimanual techniques were safe and feasible for clipping ruptured aneurysms, which not only provided additional information and a better view of the regional anatomical features but also avoided unnecessary manipulation and retraction.

**Table 1:** The clinical features of all patients.

Case	Gender	Age (years)	Location	Rupture	Hematoma	H-Hgrade	Fisher grade	KPS	
								Pre	Post
1	M	70	MCA	Y	Y	2	2	70	100
2	M	75	ACA	Y	Y	4	4	20	60
3	M	67	AComA	Y	N	3	3	70	100
4	M	45	AComA	Y	N	2	3	70	100
5	M	74	AComA	Y	N	3	3	40	90
6	M	56	OphthA	Y	N	3	3	50	100
7	F	62	ACA	Y	Y	4	4	30	90
8	F	64	PComA	Y	N	3	4	60	80

ACA, anterior cerebral artery; AComA, anterior communicating artery; MCA, middle cerebral artery; OphthA, ophthalmic artery; PComA, posterior communicating artery.

In the current study, one typical case with preoperative and postoperative imaging and intraoperative findings is highlighted.

**Figure 1:**
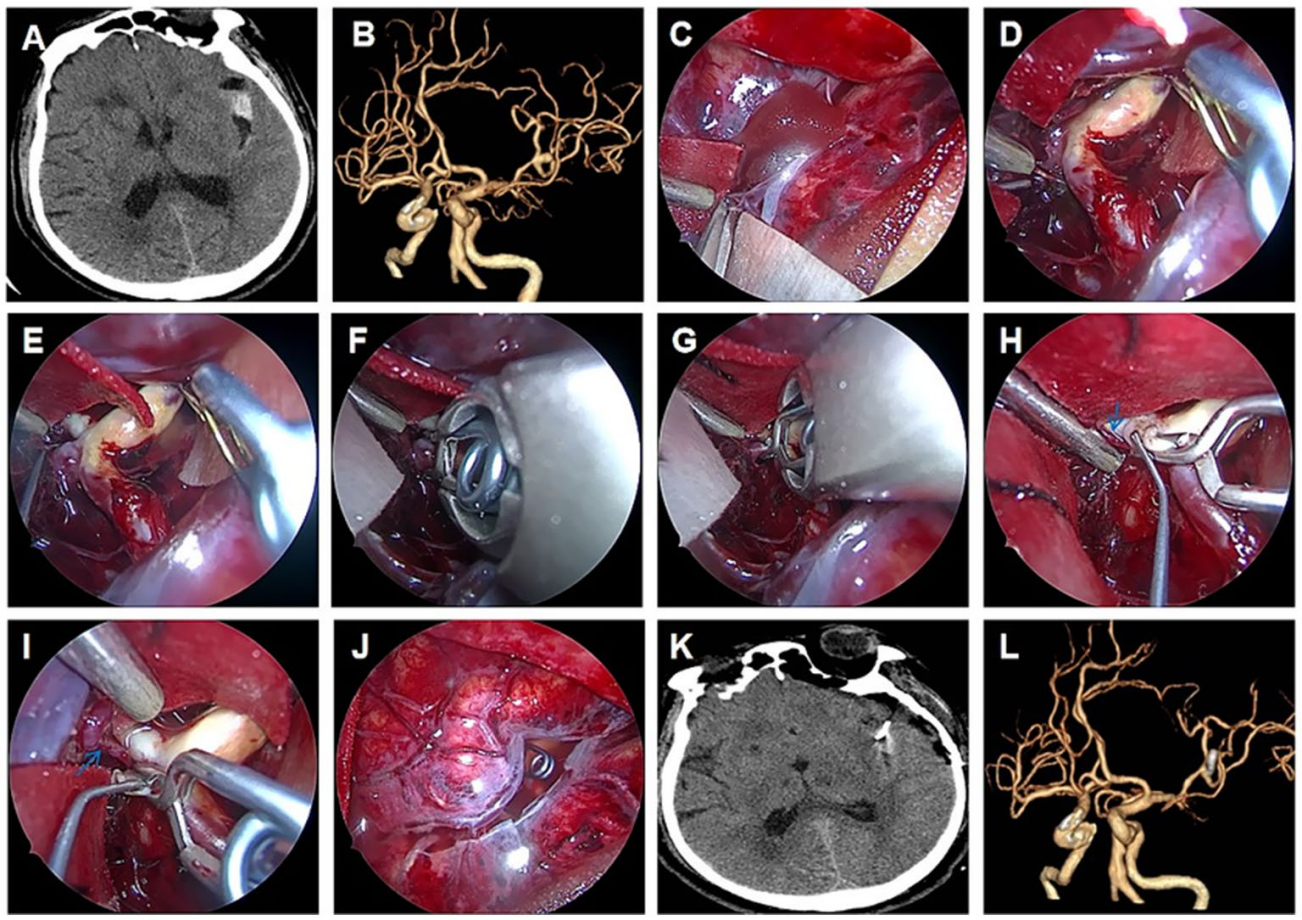
Endoscopic clipping of MCA aneurysm demonstrating retractorless exposure. Emergent CT demonstrated SAH **(A)** and a subsequent MCA aneurysm on CTA **(B)**. Fully endoscopic opening of the Sylvian fissure **(C)**. Fully endoscopic exposure of the ruptured aneurysm **(D,E)**. Fully endoscopic dissection and clipping of an aneurysm **(F,G)**. Post-clipping inspection indicated complete occlusion of the aneurysm and preservation of the branch artery and perforators (arrow) **(H,I)**. No injury of the blind area posterior to the head of the lens **(J)**. None of the postclipping cerebral infarctions occurred on CT **(K)**, and CTA before dismission confirmed complete occlusion of the aneurysm **(L)**.

Case 1 was a 70-year-old man with a serious headache for 5 days. Emergent CT demonstrated SAH (Figure 1A), and subsequent CTA (Figure 1B) revealed an MCA aneurysm. He was administered emergent aneurysm clipping using fully endoscopic techniques. After a minimally invasive keyhole craniotomy, the Sylvian fissure was opened using fully endoscopic techniques (Figure 1C). Further bimanual dissection was performed to expose the ruptured aneurysm using retractorless techniques under an endoscopic view (Figures 1D,E). After adequate dissection, the aneurysm was clipped using fully endoscopic bimanual techniques (Figures 1F,G). Post-clipping inspection indicated complete occlusion of the aneurysm and preservation of the branch artery and perforators (arrow) (Figures 1H,I). There was no injury to the blind area posterior to the head of the lens (Figure 1J). None of the post-clipping cerebral infarctions occurred on CT (Figure 1K), and CTA before discharge confirmed the complete occlusion of the aneurysm (Figure 1L). The patient achieved favorable recovery.

## Discussion

The fully endoscopic bimanual technique, termed “endoscopic controlled operation,” represents a significant advancement in neurosurgical mode, offering a minimally invasive and more accurate alternative to traditional microscopic neurosurgery. In this study, we present our initial experience with the application of transcranial fully endoscopic bimanual techniques for clipping ruptured aneurysms, which yielded satisfactory outcomes. The findings suggest that fully endoscopic clipping of aneurysms is feasible and may contributes to reducing the incidence of intraprocedural rupture and preserving patent and perforating vasculature. To our knowledge, this is the first series that focuses on the application of fully endoscopic techniques in the treatment of ruptured aneurysms.

### Comparison between routine clipping under microscopic view and endoscopic clipping

Although considerable effort has been made to improve the outcome, clipping a ruptured aneurysm remains a challenging and demanding task. Even in the most experienced hands, there still exist several major concerns about clipping ruptured aneurysms: unexpected residual filling of clipped aneurysms, compromising the parent or branching artery, and intraprocedural rupture of aneurysms. Accumulating studies have demonstrated that all these concerns are ascribed to the compromise of identification of the relationship between the aneurysm and the surrounding arteries before, during, and after clipping under a microscopic view. Several authors have attempted to use intraoperative Indocyanine Green video angiography (ICG-VA) to improve aneurysm clipping via a microscope (9). However, under a conventional microscope view, this is not always possible because of the straight line of the view imposed by the microscope and the shielding of the brain tissue and vasculature. In order to gain a better view of the backside and formerly hidden corners, the neurosurgeon has to perform more retraction of the brain and displace the vascular structures and even the aneurysm. All these manipulations are believed to improve the incidence of the intraprocedural rupture of the aneurysms and the injury of the neurovascular structure (10).

Recently, the advantages of endoscopes in neurosurgery have been well established, including a brighter illumination in the depth of the surgical field, an extended viewing angle, and detailed patho-anatomical information in a close-up fashion. Growing evidence has demonstrated that endoscopic procedures have become the finest workhorses of contemporary neurosurgery, providing more detailed features and extending the neurosurgeon’s equipment and surgical options (11, 12). A study has demonstrated that endoscopic inspection before clipping might reduce overexposure and mobilization of the aneurysm, which favors reducing the incidence of intraprocedural rupture (13). Endoscope-assisted clipping of aneurysms also helped significantly reduce unattended parent artery, branch, or perforator occlusion (12). Massimo Gallieni et al. found a total of 208 aneurysms clipping under microscopic view; the endoscopic inspection allowed for detection of neck remnant in 22 cases, branch occlusion in 11 cases, and perforator occlusion in 9 cases (14). Kang U Kim et al. demonstrated the clinical significance of endoscope-assisted clipping in preventing post-clipping cerebral infarction from 15.7% before applying endoscopic assistance to 6.9% after endoscope application (15). Evidence has demonstrated that endoscope-assisted clipping can provide additional information beyond the microscope and ICG and change the operative plan, including residual aneurysm and incomplete clipping, which are negative in intraoperative ICG (16). Moreover, endoscopic clipping enables minimally invasive keyhole craniotomy (13). Even in ruptured aneurysms, we still performed minimally invasive keyhole craniotomy in 37.5% of cases.

### Comparison between endoscopic-assisted clipping and fully endoscopic clipping

Although the role of endoscopes has shifted from endoscopically assisted operation (17) to endoscopically controlled operation in more and more neurosurgery, such as MVD (18), brain base tumor (19). However, experience with endoscopic procedures in aneurysm clipping is still limited to an adjunct to conventional microscopic surgery in several clinical retrospective studies (12, 20). Debates regarding the applicability of fully endoscopic aneurysm clipping persist in the neurosurgical community. The main concern is the disappearance of the view after the sudden rebleeding of aneurysms. We usually adopt several measures to prevent possible rupture, including proximal control and dual suction. Even with heavy bleeding, which results in temporary loss of visualization, we can keep the endoscope tip clean via rapid irrigation and subsequent suction. As a matter of fact, growing evidence has demonstrated that endoscopic technique could contribute to a lower rebleeding rate (12). Moreover, with accumulated endoscopic experience and skill, controlling bleeding is not always a challenge at skilled hands, and fully endoscopic techniques have gained increasing popularity in neurosurgery involving challenging vascular manipulation (21, 22).

During the procedure of endoscopic-assisted clipping, the surgeon has to move the line of sight significantly between viewing the operative field through the microscope and viewing the endoscope monitor, which is still in “outside-door” surgery mode. Therefore, incompletely clipped aneurysms and occlusion of the branch or perforator were identified on follow-up, even in the patient group that only underwent endoscopic inspection but was clipped under microscopic view (13, 23). It is important for the neurosurgeons to obtain real-time information about aneurysms hidden from the microscopic view during the entire procedure. To do this, we attempted fully endoscopic bimanual techniques and obtained clear real-time details of aneurysms and relative vessels before, during, and after clipping in all cases. With the help of the advantage of “indoor surgery mode,” our initial experience recorded total occlusion of the aneurysms without any compromise of the parent artery and branch artery in all patients. The results demonstrate the feasibility of fully endoscopic techniques during the whole procedure of aneurysm clipping. A more real-time and clear view of the aneurysm can be obtained, which greatly improves the accuracy of the manipulation and contributes to decreasing the intraprocedural rupture. The “indoor surgery mode” afforded by fully endoscopic techniques allows surgeons to look around corners, enabling direct inspection of aneurysm necks and adjacent perforators with minimal retraction. No intraprocedural rupture occurred in our series, and no infarction was recorded after surgery. All results in this study are superior to those of previous reports using clipping under a microscope or even endoscope assistance. Moreover, compared with the previous clipping of aneurysms via the endoscopic endonasal approach (24), we believe it is easier to perform temporary clipping via the transcranial approach, which was found to contribute to decreasing intraprocedural rupture in our experience.

Some authors have suggested that endoscopic indications for aneurysm clipping are unruptured aneurysms (25). However, the initial experience demonstrates that ruptured aneurysms do not contraindicate fully endoscopic techniques. In our opinion, with the help of high-quality visualization of fine structures under an endoscope, more meticulous manipulation can be achieved, which favors perfect clipping of a ruptured aneurysm.

There are also other tips that we believe contribute to the safety of endoscopic clipping of ruptured aneurysms. Usually, we cover the blind area posterior to the head of the lens with a sterile plastic pad to avoid injury, and with accumulating experience, the surgeon can establish enough spatial feeling to go into and out of the surgical field freely without concerns about injury. Complications in direct relation to the endoscope were not observed in our series. Warming the endoscope before use is another useful skill to prevent fogging of the lens. During surgery, we also used a controlled endoscopic suction and irrigation sheath to keep the endoscope tip clean. We believe that all these skills ensure the safety and feasibility of fully endoscopic clipping of aneurysms.

### Limitations

This study aims to demonstrate the safety and feasibility of fully endoscopic clipping of ruptured aneurysms. The advantages of fully endoscopic clipping were observed. Nevertheless, there are certain limitations that warrant attention and further investigation. First, the limited number of cases in this study hinders a comparative analysis with clipping aneurysms under microscopic view. In the future, larger-scale randomized controlled trials will yield more compelling conclusions. Second, the learning curve of fully endoscopic techniques should be acknowledged, which requires adequate practice and training. With the development of specialized equipment and 3-D techniques in the future, we envision that fully endoscopic clipping will emerge as a significant component of aneurysm treatment.

## Conclusion

This study presents a case series of ruptured aneurysms treated with fully endoscopic clipping. In this approach, the neurosurgeon not only acquired valuable information for decision-making during surgery but also avoided unnecessary manipulation and retraction. This study highlights the advantages of fully endoscopic techniques and demonstrates that clipping ruptured aneurysms using fully endoscopic techniques is safe and feasible. We believe that with accumulating experience and skill, the benefits derived from fully endoscopic techniques might further the quality of ruptured aneurysm treatment.

## Data Availability

The original contributions presented in the study are included in the article/supplementary material, further inquiries can be directed to the corresponding author.
